# Strain-Tunable Visible-Light-Responsive Photocatalytic Properties of Two-Dimensional CdS/g-C_3_N_4_: A Hybrid Density Functional Study

**DOI:** 10.3390/nano9020244

**Published:** 2019-02-12

**Authors:** Guangzhao Wang, Feng Zhou, Binfang Yuan, Shuyuan Xiao, Anlong Kuang, Mingmin Zhong, Suihu Dang, Xiaojiang Long, Wanli Zhang

**Affiliations:** 1 School of Electronic Information Engineering, Key Laboratory of Extraordinary Bond Engineering and Advanced Materials Technology of Chongqing, Yangtze Normal University, Chongqing 408100, China; zhoufeng9966@126.com (F.Z.); dangsuihu@126.com (S.D.); longxiaojiang@yznu.edu.cn (X.L.); zhangwl@yznu.cn (W.Z.); 2 School of Chemistry and Chemical Engineering, Yangtze Normal University, Chongqing 408100, China; 3 Institute for Advanced Study, Nanchang University, Nanchang 330031, China; syxiao@hust.edu.cn; 4 School of Physical Science and Technology, Southwest University, Chongqing 400715, China; zhongmm@swu.edu.cn

**Keywords:** CdS/g-C_3_N_4_, strain-tunable, photocatalysis, water splitting, hybrid density functional

## Abstract

By means of a hybrid density functional, we comprehensively investigate the energetic, electronic, optical properties, and band edge alignments of two-dimensional (2D) CdS/g-C${}_{3}$N${}_{4}$ heterostructures by considering the effect of biaxial strain and pH value, so as to improve the photocatalytic activity. The results reveal that a CdS monolayer weakly contacts with g-C${}_{3}$N${}_{4}$, forming a type II van der Waals (vdW) heterostructure. The narrow bandgap makes CdS/g-C${}_{3}$N${}_{4}$ suitable for absorbing visible light and the induced built-in electric field between the interface promotes the effective separation of photogenerated carriers. Through applying the biaxial strain, the interface adhesion energy, bandgap, and band edge positions, in contrast with water, redox levels of CdS/g-C${}_{3}$N${}_{4}$ can be obviously adjusted. Especially, the pH of electrolyte also significantly influences the photocatalytic performance of CdS/g-C${}_{3}$N${}_{4}$. When pH is smaller than 6.5, the band edge alignments of CdS/g-C${}_{3}$N${}_{4}$ are thermodynamically beneficial for oxygen and hydrogen generation. Our findings offer a theoretical basis to develop g-C${}_{3}$N${}_{4}$-based water-splitting photocatalysts.

## 1. Introduction

Gaining hydrogen through photocatalytic water splitting by use of solar energy provides a new way to solve the problems of energy shortage and environmental pollution. A large number of semiconductors, such as TiO${}_{2}$ [1], ZnO [2], KNbO${}_{3}$ [3], and NaNbO${}_{3}$ [4] have drawn much attention as promising photocatalysts, but they can merely utilize ultraviolet light, which only makes up only 4% of solar energy. Some photocatalysts, such as bulk CdS [5], have suitable bandgaps for visible light absorption, but lacks stability due to the self-oxidation of photogenerated species. Thus, it is challenging to find efficient water-splitting photocatalysts, and some appropriate strategies should be taken to modulate the electronic and photocatalytic properties of pristine photocatalysts. Generally, introduction of dopants [6,7], loading noble metal [8], dye sensitizing [9] and cocatalysis through constructing heterojunctions [10,11,12] are effective at improving the photocatalytic activity. The desired photocatalyst must have the conduction band minimum (CBM) and valence band maximum (VBM) individually above the water reduction (H${}^{+}$/H${}_{2}$) potential and below the water oxidation (O${}_{2}$/H${}_{2}$O) potential. Besides, the theoretical minimum bandgap of 1.23 eV is required for water splitting [13] considering the overpotential accompanied by water redox processes.

Since graphene was prepared, 2D materials including hexagonal boron nitride [14], graphite-like zinc oxide [15], transition-metal dichalcogenides [16], and MXenes [17] have been extensively investigated and utilized in the area of optoelectronics and photocatalysts. Particularly, the graphite-like carbon nitride (g-C${}_{3}$N${}_{4}$) is a prospective photocatalyst used for hydrogen generation by photocatalytic decomposition of water [18]. g-C${}_{3}$N${}_{4}$ has a suitable bandgap of 2.7 eV for visible light absorption. However, g-C${}_{3}$N${}_{4}$ exhibits poor photocatalytic efficiency because of the fast recombination of photogenerated electron–hole pairs [19,20,21]. This factor obviously restrains the photocatalytic efficiency of g-C${}_{3}$N${}_{4}$. It is of great significance to adopt measures to regulate the electronic structures of g-C${}_{3}$N${}_{4}$ in a bid to enhance the photocatalytic performance. Especially, a large number of 2D heterostructures, such as ZnO/WS${}_{2}$ [22], AlN/WS${}_{2}$ [23], GaN/WS${}_{2}$ [24], g-C${}_{6}$N${}_{6}$/g-C${}_{3}$N${}_{4}$ [25], g-C${}_{3}$N${}_{4}$/MoS${}_{2}$ [26] and g-C${}_{3}$N${}_{4}$/C${}_{2}$N [27] exhibit significantly improved photocatalytic activity as compared to pristine 2D materials. In these heterostructures, the formed built-in electric field caused by the charge accumulation/depletion around the interfaces promotes the effective separation and migration of photogenerated carriers, which is beneficial to enhance the photocatalytic performance. A recent theoretical study [28] reports the stability, electronic structures, and offset of 2D CdS/g-C${}_{3}$N${}_{4}$ heterostructure, and the result suggests that the heterostructure has suitable bandgap and band alignments for visible light photocatalytic water splitting. Moreover, the induced electric field between CdS layer and g-C${}_{3}$N${}_{4}$ also accelerates the separation of photogenerated carriers and improves the photocatalytic activity. However, whether the biaxial strain will improve the photocatalytic activity of CdS/g-C${}_{3}$N${}_{4}$ is still unclear. Besides, it is also unclear whether the photocatalytic activity of CdS/g-C${}_{3}$N${}_{4}$ is affected by the pH of electrolyte. These two problems have to be solved in a bid to obviously enhance the photocatalytic performance of CdS/g-C${}_{3}$N${}_{4}$.

The purpose of this work is to investigate the energetic, electronic, optical property and band edge alignments of CdS/g-C${}_{3}$N${}_{4}$ as well as the effect induced by the biaxial strain and the pH of electrolyte, in order to regulate the photocatalytic performance. This work is organized as follows. Section 2 depicts the computational details, while Section 3 displays the results and discussion about the energetic, optical, optical, band edge alignments as well as the photocatalytic property of CdS/g-C${}_{3}$N${}_{4}$ heterostructure with the consideration of biaxial strain and pH, and ultimately Section 4 lists some concluding remarks.

## 2. Computational Details

The CdS/g-C${}_{3}$N${}_{4}$ heterostructure, which consists of 3 Cd, 3 S, 6 C, and 8 N atoms, is constructed through vertically stacking a $\sqrt{3}\times \sqrt{3}$ supercell of hexagonal CdS single-layer on a 1×1 g-C${}_{3}$N${}_{4}$ cell. We carry out density functional theory (DFT) calculations by means of the general gradient approximation (GGA) [29] of Perdew–Burke–Ernzerhof (PBE) [30] and hybrid density functional of HSE06 [31], as implemented in the Vienna ab initio simulation package (VASP) [32]. We adopt the projected-augmented-wave (PAW) method [33] to describe the electron-ion interaction and DFT-D3 correction [34] to well treat long-range vdW interaction. To avoid the interactions introduced by the periodic structures, a vacuum of 18 Å is used. We first optimize the geometries by use of PBE, and then accurately calculate the electronic and optical properties by utilization of HSE06. The plane-wave cutoff energy is set as 500 eV, and a Monkhorst-pack [35] k-point mesh of 13×13×1 for CdS cell, 9×9×1 for g-C${}_{3}$N${}_{4}$ cell and CdS/g-C${}_{3}$N${}_{4}$ heterostructures are used. All the structures are fully relaxed until the energy and force on each atom are individually reduced to 10${}^{-5}$ eV and 0.02 eV/Å. The valence electron configurations of of Cd (4d${}^{10}$5s${}^{2}$), S (3s${}^{2}$3p${}^{4}$), C(2s${}^{2}$2p${}^{2}$), and N (2s${}^{2}$2p${}^{3}$) are considered to construct the PAW potentials.

Finally, the optical absorption spectra of g-C${}_{3}$N${}_{4}$ and CdS/g-C${}_{3}$N${}_{4}$ composite is calculated by use of HSE06. The absorption coefficient is obtained from the the real and imaginary parts of the frequency dependent complex dielectric function ε(ω) = $\varepsilon_{1}\left(\omega \right)$ + $i\varepsilon_{2}\left(\omega \right)$ according to the following relationship [36]

(1) $$I\left(\omega \right)=\sqrt{2}\omega \sqrt{\sqrt{\varepsilon_{1}^{2}\left(\omega \right)+\varepsilon_{2}^{2}\left(\omega \right)}-\varepsilon_{1}\left(\omega \right)}$$

The imaginary part of the dielectric function $\varepsilon_{2}$ is calculated as [37]

(2) $$\varepsilon_{2}\left(\hbar \omega \right)=\frac{2e^{2}\pi }{\Omega \varepsilon_{0}}\sum_{k,v,c}|\langle \psi_{k}^{c}\left|u\cdot r\right|\psi_{k}^{v}{\rangle |}^{2}\delta \left(E_{k}^{c}-E_{k}^{v}-\hbar \omega \right)$$

where Ω, *v*, *c*, ω, u, $\psi_{k}^{v}$ and $\psi_{k}^{c}$ denotes the unit-cell volume, valence bands, conduction bands, photon frequencies, the vector defining the polarization of the incident electric field, the occupied and unoccupied wave functions at point *k* in reciprocal space, respectively, while the real part of the dielectric function $\varepsilon_{1}$ can be obtained from imaginary part $\varepsilon_{2}$ by the Kramer-Kronig relationship [38].

(3) $$\varepsilon_{1}\left(\omega \right)=1+\frac{2}{\pi }p\int_{0}^{\infty }\frac{\varepsilon_{2}\left(\omega^{{}^{\prime }}\right)\omega^{{}^{\prime }}}{\omega^{{}^{\prime }2}-\omega^{2}}d\omega^{{}^{\prime }}$$

where *p* denotes the principal value of the integral.

## 3. Results and Discussion

**Figure 1:**
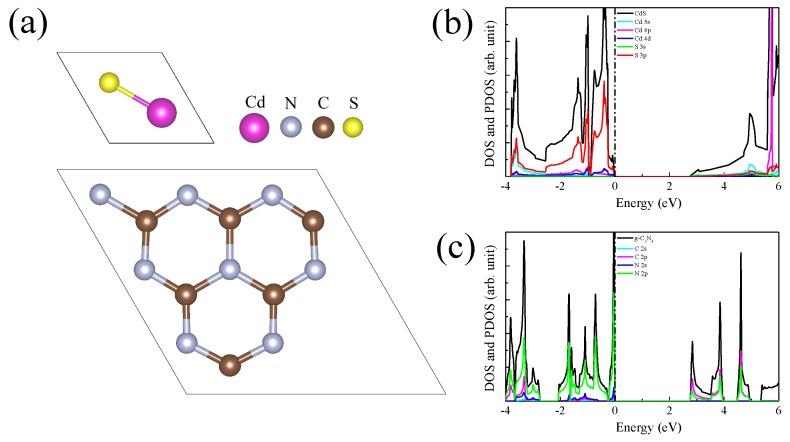
(**a**) Crystal structures of CdS single-layer and g-C${}_{3}$N${}_{4}$. DOS and PDOS of (**b**) CdS single-layer and (**c**) g-C${}_{3}$N${}_{4}$.

The geometry structures, density of states (DOS) and projected density of states (PDOS) of CdS monolayer and g-C${}_{3}$N${}_{4}$ are depicted in Figure 1. The calculated lattice constants for CdS and g-C${}_{3}$N${}_{4}$ single-layers are respectively a = b = 4.245 and a = b = 7.134 Å, and the obtained bandgaps for CdS and g-C${}_{3}$N${}_{4}$ single-layers are, respectively, 2.74 and 2.77 eV, which are well consistent with previous experiment and theoretical reports [28]. The VBM of CdS single-layer mainly consists of S 3p, Cd 4d and Cd 4p orbitals, whereas the CBM is primarily contributed by Cd 5s character. For g-C${}_{3}$N${}_{4}$, the VBM is mainly composed of N 2p orbitals with some amount of C 2p and N 2s orbitals, while the CBM is comprised of C 2p and N 2p characters.

**Figure 2:**
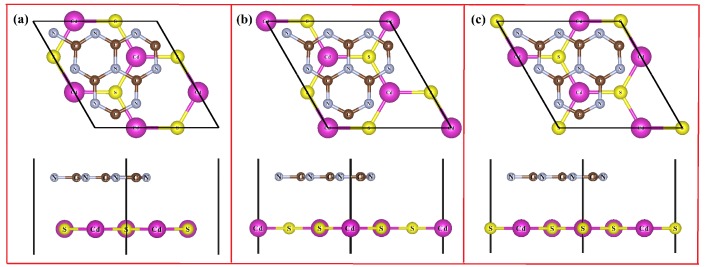
Top and side views of three possible stackings of CdS/g-C${}_{3}$N${}_{4}$ heterostructures.

The lattice mismatch is defined as: $[\left(L_{g-C_{3}N_{4}}-L_{s-\mathrm{CdS}}\right)/L_{s-\mathrm{CdS}}]\times 100\%$, where $L_{g-C_{3}N_{4}}$ and $L_{s-\mathrm{CdS}}$ are the lattice constants of g-C${}_{3}$N${}_{4}$ cell and $\sqrt{3}\times \sqrt{3}$ CdS supercell, respectively. When a $\sqrt{3}\times \sqrt{3}$ CdS supercell contacts with a 1×1 g-C${}_{3}$N${}_{4}$ cell, the lattice mismatch is only −2.97%, which is good for the construction of CdS/g-C${}_{3}$N${}_{4}$ heterostructure. We consider a $\sqrt{3}\times \sqrt{3}$ CdS supercell with with tree special rotation angles of 0${}^{\circ }$, 120${}^{\circ }$, and 240${}^{\circ }$ sitting on a 1×11g-C${}_{3}$N${}_{4}$ cell with fixed angles to construct three possible configurations of CdS/g-C${}_{3}$N${}_{4}$, as depicted in Figure 2. These different heterostructures are call as CdS/g-C${}_{3}$N${}_{4}$ (i), (ii), and (iii), respectively. The optimized lattice constants for CdS/g-C${}_{3}$N${}_{4}$ (i), (ii) and (iii) are respectively 6.954, 6.955 and 6.920 Å, slightly smaller than the lattice of g-C${}_{3}$N${}_{4}$. This may be attributed to the atom rearrangements in the heterostructures. The obtained bandgaps for CdS/g-C${}_{3}$N${}_{4}$ (i), (ii) and (iii) are 2.745, 2.746 and 2.676 eV, respectively. Though the bandgaps of these heterostructures are almost the same as the bandgap of g-C${}_{3}$N${}_{4}$, the absorption of visible light is significantly improved. This will be detailed in the following discussion.

**Figure 3:**
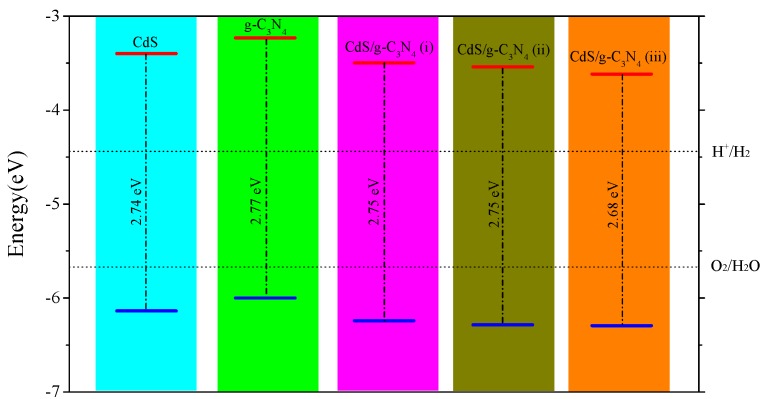
Band edge alignments for CdS single-layer, g-C${}_{3}$N${}_{4}$, CdS/g-C${}_{3}$N${}_{4}$ (i), (ii), and (iii) in contrast with water redox levels.

To explore the thermodynamic stability, the interface binding energy ($E_{b}$) is calculated according to the following relationship:

(4) $$E_{b}=E_{\mathrm{CdS}/g-C_{3}N_{4}}-E_{\mathrm{CdS}}-E_{g-C_{3}N_{4}}$$

where $E_{\mathrm{CdS}/g-C_{3}N_{4}}$, $E_{\mathrm{CdS}}$, and $E_{g-C_{3}N_{4}}$ denote the total energies of CdS/g-C${}_{3}$N${}_{4}$ heterostructure, CdS single-layer, and g-C${}_{3}$N${}_{4}$, respectively. The $E_{b}$ values for CdS/g-C${}_{3}$N${}_{4}$ (i), (ii) and (iii) are respectively −1.62633, −1.62548 and −1.62630 eV, implying these heterostructures are exothermic and are energetically favorable. Besides, the differences of $E_{b}$ among these structures are so small that these three configurations may be experimentally prepared at the same time. These three configurations have similar energy values. Furthermore, the band alignments depicted in Figure 3 also indicate that the band edge positions of these three heterostructures are close. Thus, our discussion is mainly focused on the CdS/g-C${}_{3}$N${}_{4}$ (i). The interface adhesion energy ($E_{a}$) is calculated according to the following equation:

(5) $$E_{a}=E_{b}/S$$

where *S* is the area of CdS/g-C${}_{3}$N${}_{4}$ heterostructure vertical to the vacuum direction. The $E_{a}$ for CdS/g-C${}_{3}$N${}_{4}$ (i) is −19.4 meV/Å${}^{2}$, within the scope of typical vdW heterostructure of −20 meV/Å${}^{2}$ [39].

As an ideal water-splitting photocatalyst, its band edges must be located in proper positions. The CBM and VBM must straddle the water redox potentials to satisfy the thermodynamic criterion for overall water splitting. Figure 3 displays the band edge alignments for CdS monolayer, g-C${}_{3}$N${}_{4}$, CdS/g-C${}_{3}$N${}_{4}$ (i), (ii) and (iii). The band edges of these systems are all straddle the water redox levels, which is propitious to spontaneously produce both hydrogen and oxygen.

**Figure 4:**
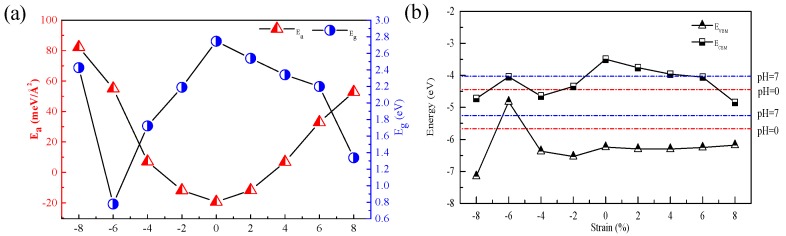
(**a**) Varied interface adhesion energies ($E_{a}$) and bandgaps ($E_{g}$) of CdS/g-C${}_{3}$N${}_{4}$ heterostructures with different biaxial strains. (**b**) Band edge alignments of CdS/g-C${}_{3}$N${}_{4}$ heterostructures with different biaxial strains. The red and blue horizontal lines are the water redox potentials as pH = 0 and pH = 7, respectively.

The appearance of strain can not be ignored due to the lattice mismatch between different 2D semiconductors. It is found that for 2D materials, the electronic and optical properties can be modulated through strain engineering [40,41,42]. We consider the influence caused by both tensile and compressive biaxial strain on the energetic, electronic, and photocatalytic properties of CdS/g-C${}_{3}$N${}_{4}$. The biaxial strain is defined as $\epsilon =[\left(a-a_{0}\right)/a_{0}]\times 100\%$, in which *a* and $a_{0}$ are the lattice parameters of strained and pristine CdS/g-C${}_{3}$N${}_{4}$ heterostructures, respectively. ϵ<0 means the structure is compressed, while ϵ>0 means the structure is stretched. Figure 4 gives the varied $E_{a}$ and $E_{g}$ values of CdS/g-C${}_{3}$N${}_{4}$ heterostructures of different biaxial strain with 2% apart. The $E_{a}$ value gets smaller within the scope of ϵ = −8% to ϵ = 0 but gets larger in the range of ϵ = 0 to ϵ = 8%. The unstrained CdS/g-C${}_{3}$N${}_{4}$ heterostructure has the least interface adhesion energy, which implies that unstrained configuration has advantage in energy in contrast with strained configuration. The calculated $E_{a}$ value with the ϵ in the range from −8% to 8% are 82.2, 55.1, 7.1, −11.8, −19.4, −11.7, 6.8, 32.9 and 52.8 meV/Å${}^{2}$, indicating the formation of the heterostructures with the ϵ = −2%, 0, 2% are exothermic. The $E_{g}$ value decreases in the range of ϵ = −8% to ϵ = −6%, increases in the range of ϵ = −6% to ϵ = 0, and decreases in the range of ϵ = 0 to ϵ = 8%. This suggests that the visible light absorption can be modulated by tuning the bandgaps through biaxial strain engineering. The unstrained heterostructure has the largest bandgap. The obtained bandgaps for CdS/g-C${}_{3}$N${}_{4}$ heterostructures in the range of ϵ = −8% to ϵ = 8% are 2.43, 0.78, 1.72, 2.19, 2.75, 2.54, 2.34, 2.20 and 1.34 eV.

The photocatalytic performance is affected by the pH of electrolyte. Particularly, the standard hydrogen electrode potential varies with the pH varies. The standard reduction (H${}^{+}$/H${}_{2}$) in contrast with the vacuum level is calculated by: E${}_{H^{+}/H_{2}}$ = −4.44 eV + pH × 0.059 eV [43]. With the consideration of the difference of 1.23 eV [44] between water redox potentials during the water redox reactions, the oxygen potential (O${}_{2}$/H${}_{2}$O) is calculated by: E${}_{O_{2}/H_{2}O}$= E${}_{H^{+}/H_{2}}$− 1.23 eV = −5.67 eV + pH × 0.059 eV. The method has been successfully applied to predict the photocatalytic properties of P and As doped C${}_{2}$N monolayer [45], CdS/ZnSe heterostructure [46], and (Bule P)/BSe heterostructure [47] with considering the effect of pH on the standard redox potentials with respect to the vacuum level.

The band edge alignments of CdS/g-C${}_{3}$N${}_{4}$ heterostructures with diverse biaxial strains are displayed in Figure 4. Both CBM and VBM of CdS/g-C${}_{3}$N${}_{4}$ of ϵ = −2%, 0, 2%, 4%, 6% individually straddle the water redox levels in the pH range of 0–1.6, 0–14, 0–11.5, 0–8.1, 0–6.5. In the pH range of 0–14, the VBM and CBM of CdS/g-C${}_{3}$N${}_{4}$ with ϵ = −8%, −4%, 8% are individually lower than the water oxidation (O${}_{2}$/H${}_{2}$O) and reduction (H${}^{+}$/H${}_{2}$) potentials, which means that these heterostructures are only beneficial for oxygen generation. For the case of ϵ = −6%, the VBM and CBM are individually above the water oxidation (O${}_{2}$/H${}_{2}$O) and reduction (H${}^{+}$/H${}_{2}$) potentials when the pH is lower than 6.9. When the pH is lower than 6.5, the CdS/g-C${}_{3}$N${}_{4}$ with ϵ = 0, 2%, 4%, 6% are thermodynamically feasible for over all water redox reactions, while the composites of ϵ = −2%, −6%, −8%, −4%, 8% are propitious to spontaneously generate oxygen. Therefore, adjustment of the pH lower than 6.5 is conducive to improve the photocatalytic activity of CdS/g-C${}_{3}$N${}_{4}$.

**Figure 5:**
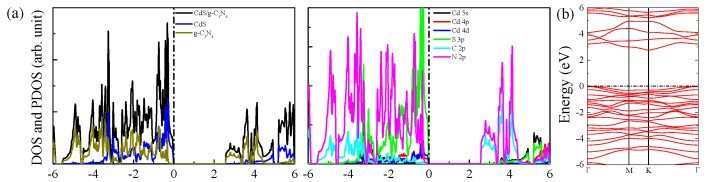
(**a**) DOS, PDOS and (**b**) band structures of CdS/g-C${}_{3}$N${}_{4}$ heterostructure.

**Figure 6:**
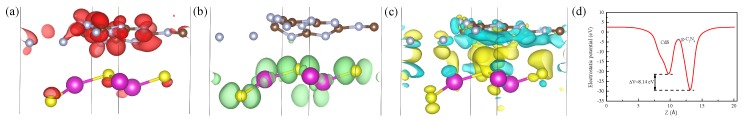
Partial charge densities of (**a**) CBM, (**b**) VBM, (**c**) the charge density difference, and (**d**) potential drop across the interface of CdS/g-C${}_{3}$N${}_{4}$ heterostructure.

Next, we plot the DOS, PDOS, and band structures of unstrained CdS/g-C${}_{3}$N${}_{4}$ to shed light on the physical mechanism of water splitting of CdS/g-C${}_{3}$N${}_{4}$. Figure 5 shows that the CBM and VBM are individually donated by g-C${}_{3}$N${}_{4}$ and CdS layer, suggesting that the CdS/g-C${}_{3}$N${}_{4}$ is a type II heterostructure. The partial charge density of CBM and VBM in Figure 6 also suggests the CBM of g-C${}_{3}$N${}_{4}$ is predominately contributed by g-C${}_{3}$N${}_{4}$ and the VBM is mainly donated by CdS layer. The VBM is primarily composed of S 3p, Cd 4d and Cd 4p states, while the CBM is predominately dominated by N 2p and C 2p states. Taking the electronic transition of angular momentum selection rules of △l=±1 into account, after absorbing photons, the electrons primarily migrate from Cd 4d orbitals below the Fermi level to N 2p and C 2p orbitals in conduction band.

The charge density difference of CdS/g-C${}_{3}$N${}_{4}$ heterostructure in Figure 6c, where cyan and yellow regions represent charge depletion and accumulation, respectively. It is obvious that electrons migrate from CdS layer to g-C${}_{3}$N${}_{4}$. Based on the Bader charge analysis, the transferred charge is 0.027 |e|, which is enough to introduce a large potential drop between the g-C${}_{3}$N${}_{4}$ and CdS layer. Figure 6d lists potential drop across the interface of CdS/g-C${}_{3}$N${}_{4}$ along the *Z* direction, i.e., the vacuum direction. The g-C${}_{3}$N${}_{4}$ has a deeper potential as compared to that of CdS layer, which drives electrons to migrate from CdS layer to g-C${}_{3}$N${}_{4}$. The potential drop (ΔV) across the interface is 8.14 eV, inducing a built-in electric field from the g-C${}_{3}$N${}_{4}$ to CdS layer. The formed built-in electric field can promote the shifts of photogenerated carriers, thus further inhibiting the recombination of photogenerated carriers. The g-C${}_{3}$N${}_{4}$ and CdS individually pose as electron acceptor and donor. Thus, the water oxidation reaction and reduction reaction occur on the CdS layer and g-C${}_{3}$N${}_{4}$, respectively. This is beneficial for improving the photocatalytic activity.

**Figure 7:**
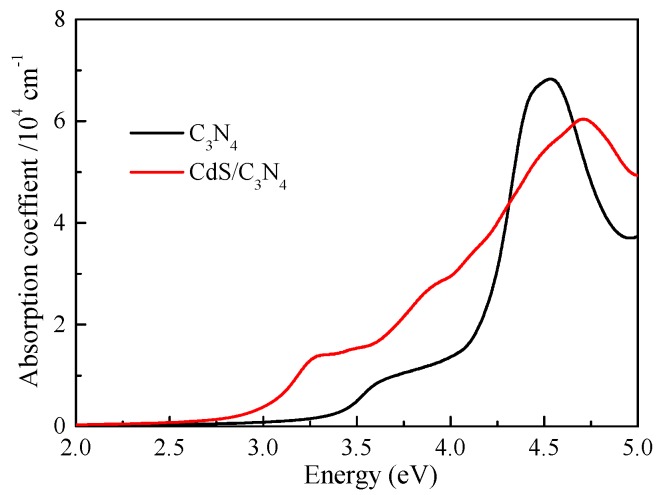
Absorption spectra of pristine g-C${}_{3}$N${}_{4}$ and CdS/g-C${}_{3}$N${}_{4}$ heterostructure.

Another key indicator to the photocatalytic performance is the optical absorption. Figure 7 depicts the obtained absorption cures for g-C${}_{3}$N${}_{4}$ and CdS/g-C${}_{3}$N${}_{4}$, the original g-C${}_{3}$N${}_{4}$ only exhibits a obvious absorption above 3.0 eV and there is almost no visible light absorption ability for g-C${}_{3}$N${}_{4}$, which may be due to the fact that only a small amount of electron density migrates electron migrates from N 2s states of valence band to C 2p and N 2p states of conduction band (see Figure 1). The adsorption edge of CdS/g-C${}_{3}$N${}_{4}$ shifts to 2.7 eV, especially the g-C${}_{3}$N${}_{4}$ shows stronger light absorption than g-C${}_{3}$N${}_{4}$ in the range of 2.7–4.3 eV, i.e., the CdS/g-C${}_{3}$N${}_{4}$ owns a broad absorption in both ultraviolet and visible light regions. According to Figure 1 and Figure 5, the reason of enhancement of light absorption should be that the electron migration from the Cd 4d states below the Fermi level to C 2p and N 2p states are significantly enhanced as compared to pristine g-C${}_{3}$N${}_{4}$.

## 4. Conclusions

In summary, the hybrid density functional HSE06 is employed to calculate the energetic, electronic and optical properties of CdS/g-C${}_{3}$N${}_{4}$, whilst taking into account different biaxial strains as well as the pH of electrolyte, in a bid to tune the photocatalytic activity of CdS/g-C${}_{3}$N${}_{4}$. When the interaction between single-layer CdS and g-C${}_{3}$N${}_{4}$, the vdW CdS/g-C${}_{3}$N${}_{4}$ heterostructure is easy to form, as the interface adhesion formation energy is negative. The predicted bandgaps and optical absorptions indicate the CdS/g-C${}_{3}$N${}_{4}$ heterostructure can absorb visible light. Furthermore, the formed built-in electric field around the interface region is helpful to accelerate electron–hole recombination. The bandgaps, interface adhesion energies, and band edge alignments in reference to water redox potentials are visibly affected by the biaxial strains. The photocatalytic performance of CdS/g-C${}_{3}$N${}_{4}$ can be modulated by tuning the biaxial strains and pH. When pH is lower than 6.5, the band edge positions of CdS/g-C${}_{3}$N${}_{4}$ are thermodynamically favorable for spontaneously producing of oxygen and hydrogen. In general, CdS/g-C${}_{3}$N${}_{4}$ is a perspective water-splitting photocatalyst.
